# Complete Genome Sequence of a Class I Newcastle Disease Virus Strain Isolate from a Breeding Chicken Flock in Sichuan, China

**DOI:** 10.1128/MRA.00337-19

**Published:** 2019-05-02

**Authors:** Shu-yun Li, Guo-jin You, Li-jing Du, Wen-wen Li, Yue-yue Liu, Ji-teng Du, Jing Xia, Yong Huang

**Affiliations:** a College of Veterinary Medicine, Sichuan Agricultural University, Wenjiang, Chengdu, Sichuan, People’s Republic of China; University of Maryland School of Medicine

## Abstract

A Newcastle disease virus (NDV) strain, APMV-1/Chicken/China(SC)/PT3/2016, was isolated from asymptomatic chickens at a breeding farm in China. The PT3 strain has a genome length of 15,198 nucleotides and is classified as subgenotype 1b of class I. Pathogenicity tests demonstrated that PT3 is a lentogenic strain.

## Abstract (precis)

A Newcastle disease virus (NDV) strain, APMV-1/Chicken/China(SC)/PT3/2016, was isolated from asymptomatic chickens at a breeding farm in China. The PT3 strain has a genome length of 15,198 nucleotides and is classified as subgenotype 1b of class I.

## ANNOUNCEMENT

Newcastle disease (ND) is one of the most deadly diseases that affect poultry, and the causative agent of ND is Newcastle disease virus (NDV) (1). NDV belongs to the genus *Avulavirus* of the family *Paramyxoviridae* and has a negative-stranded RNA genome with six transcriptional units (3′-NP-P-M-F-HN-L-5′). Phylogenetic analysis of the fusion (F) protein gene shows that NDV strains can be grouped into two different classes (I and II) within a single serotype (1). Class II could be divided into at least 18 genotypes (I to XVIII) and contains both vaccine viruses and virulent viruses found in poultry and wild birds (2, 3). Class I NDV strains, on the other hand, could be divided into three subgenotypes (1a, 1b, and 1c) and are mostly low-virulence strains, isolated from waterfowl, wild birds, and birds from live poultry markets (2, 3). Some scholars suggest that class I NDV strains have the potential to evolve into virulent strains after their circulation in chickens after acquiring mutations in the F and hemagglutinin-neuraminidase (HN) proteins (4, 5). Therefore, the isolation and pathotype identification of class I NDV strains in birds will help to monitor the evolution of NDV and should be an ongoing effort.

As a requirement of an ND surveillance program in breeding chicken farms in Sichuan, China, oropharyngeal and cloacal swabs were collected from healthy chickens and inoculated into the allantoic cavity of 9- to 10-day-old specific-pathogen-free (SPF) chicken embryos for virus isolation. An NDV strain, APMV-1/Chicken/China(SC)/PT3/2016 (here referred to as PT3), was isolated as confirmed with a hemagglutination inhibition (HI) assay. Viral RNA was extracted from fresh allantoic fluid with TRIzol, according to the manufacturer’s recommendation (Invitrogen, Carlsbad, CA, USA). Reverse transcription was performed with the reverse transcriptase (RT) kit (TaKaRa Bio, Inc., Dalian, China) according to the manufacturer’s protocol. The genomic nucleotide identity between NDV strains of class I and class II is only about 70%, and so seven pairs of primers were specifically synthesized by Sangon Biotech Co., Ltd. (Shanghai, China) for the amplification of the internal fragment of class I NDV (Table 1). The 3′ end and 5′ end sequences of the viral genome were amplified with rapid amplification of cDNA ends (RACE) (6). The amplification, purification, and sequencing of target fragments were performed as described previously (7). The complete genome sequence of PT3 was aligned with the EditSeq program in the Lasergene package (DNASTAR, Inc., Madison, WI, USA). The phylogenetic tree of class I NDV strains was constructed with the neighbor-joining method in MEGA version 7.0.14 as described previously (3, 8). Intravenous pathogenicity index (ICPI) tests were used to assess the virulence of PT3 following the OIE manual (http://www.oie.int/en/standard-setting/terrestrial-manual).

**TABLE 1:** PT3 genome amplification primers

Primer pair	Location (nucleotides)^*a*^	Upstream primer (5′–3′)	Downstream primer (5′–3′)
P1	52–2732	TCGAAATCGCACGGGTAGAAG	TTCCTAGGTTTGCTTCCATCAC
P2	2274–4692	AATGCTAAAAAGGGCCCACCT	CCTGTGACTACTATTCCTG
P3	4284–5938	TCATTCAAGCTGGCACAC	AAGGTGGTAACTCAGGTAG
P4	5516–8471	AAGGTGGTAACTCAGGTAG	GTTTGACAAGTGGAGACGAT
P5	8193–11264	TGTTAAGCCAATCTTCTGCAC	TTGTCATTATGTTTGGTCCT
P6	11168–14565	CTCTATACCAGGAACATCGG	TGCGTGGTTTGAGTAATGTCTG
P7	13861–15140	CTCCGTGGTGTATAGAAACT	ATTTTTGCCACTATGATTCGAT

a Primer positions are listed according to the Goose/Alaska/415/91 strain genome (GenBank accession no. AB524405).

**FIG 1:**
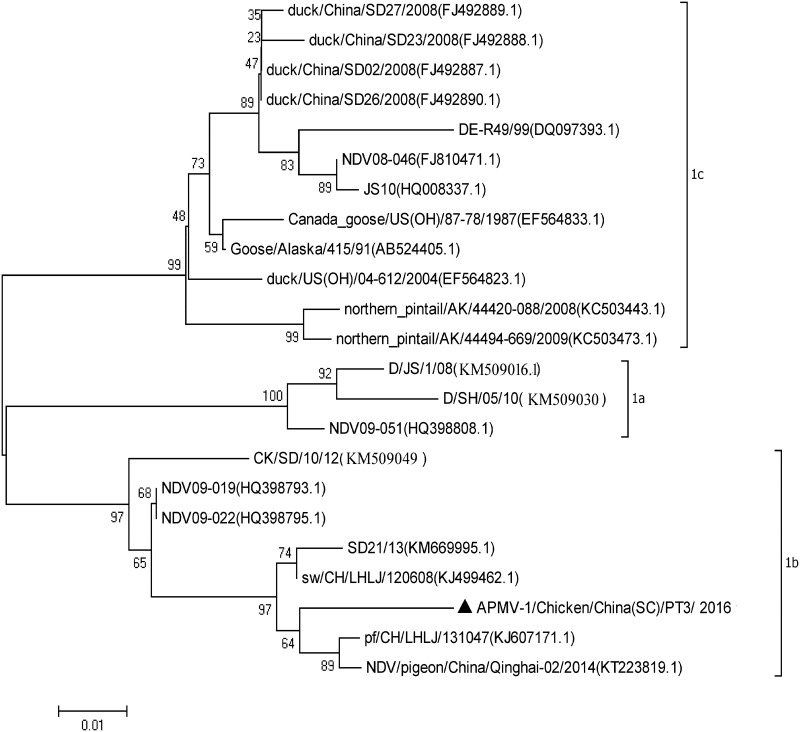
Phylogenetic tree of class I NDV strains based on the open reading frame (ORF) of F gene sequences constructed with the neighbor-joining method in MEGA version 7.0.14 and the accession numbers of the reference strains.

The complete genome sequence of PT3 was 15,198 nucleotides (nt) long. Sequence comparison showed that the antigenic determinants directly related to the fusion activity, potential *N*-glycosylation sites, and cysteine residues in the F protein are the same as those of other class I NDV strains (9). The cleavage site sequence of the F protein in PT3 was ^112^E-R-Q-E-R-L^117^, in accordance with the character of lentogenic strains. The phylogenetic tree showed that PT3 belongs to subgenotype 1b of class I (Fig. 1). A pathonenicity test showed that the ICPI value of the PT3 strain was 0.1, combined with the character of its cleavage site sequence of the F protein, indicating that PT3 is a lentogenic strain. However, since this class I NDV strain was isolated from an enclosed breeding farm rather than from wild birds, waterfowl, and live bird markets, its potential risk or role in the control of ND should be of concern.

### Data availability.

The complete genome sequence of PT3 is deposited in GenBank under the accession no. MK122776.

## References

[1] Miller PJ, Koch G 2013 Newcastle disease, other avian paramyxoviruses, and avian metapneumovirus infections, p 89–107. *In* Swayn DE (ed), Diseases of poultry, 13th ed Wiley-Blackwell Publishing, Hoboken, NJ, USA.

[2] Czeglédi A, Ujvári D, Somogyi E, Wehmann E, Werner O, Lomniczi B 2006 Third genome size category of avian paramyxovirus serotype 1 (Newcastle disease virus) and evolutionary implications. Virus Res 120:36–48. doi:10.1016/j.virusres.2005.11.009. 16766077

[3] Zhu J, Xu H, Liu J, Zhao Z, Hu S, Wang X, Liu X 2014 Surveillance of avirulent Newcastle disease viruses at live bird markets in eastern China during 2008–2012 reveals a new sub-genotype of class I virus. Virol J 11:1–9. doi:10.1186/s12985-014-0211-2. 25471313 PMC4261539

[4] Meng C, Qiu X, Yu S, Li C, Sun Y, Chen Z, Liu K, Zhang X, Tan L, Song C, Liu G, Ding C 2016 Evolution of Newcastle disease virus quasispecies diversity and enhanced virulence after passage through chicken air sacs. J Virol 90:2052. doi:10.1128/JVI.01801-15. 26656697 PMC4734012

[5] Tsunekuni R, Ito H, Otsuki K, Kida H, Ito T 2010 Genetic comparisons between lentogenic Newcastle disease virus isolated from waterfowl and velogenic variants. Virus Genes 40:252. doi:10.1007/s11262-009-0427-1. 20012681

[6] Munir M, Abbas M, Khan MT, Zohari S, Berg M 2012 Genomic and biological characterization of a velogenic Newcastle disease virus isolated from a healthy backyard poultry flock in 2010. Virol J 9:46–46. doi:10.1186/1743-422X-9-46. 22340092 PMC3295720

[7] Huang Y, Wan HQ, Liu HQ, Wu YT, Liu XF 2004 Genomic sequence of an isolate of *Newcastle disease virus* isolated from an outbreak in geese: a novel six nucleotide insertion in the non-coding region of the nucleoprotein gene. Arch Virol 149:1445. doi:10.1007/s00705-004-0297-8. 15221544

[8] Diel DG, da Silva LHA, Liu H, Wang Z, Miller PJ, Afonso CL 2012 Genetic diversity of avian paramyxovirus type 1: proposal for a unified nomenclature and classification system of Newcastle disease virus genotypes. Infect Genet Evol 12:1770–1779. doi:10.1016/j.meegid.2012.07.012. 22892200

[9] Meng C, Qiu X, Jin S, Yu S, Chen H, Ding C 2012 Whole genome sequencing and biological characterization of Duck/js/10, a new lentogenic class I Newcastle disease virus. Arch Virol 157:869–880. doi:10.1007/s00705-012-1248-4. 22310996

